# Recent Development of Non-Contact Multi-Target Vital Sign Detection and Location Tracking Based on Metamaterial Leaky Wave Antennas

**DOI:** 10.3390/s21113619

**Published:** 2021-05-22

**Authors:** Yichao Yuan, Chung-Tse Michael Wu

**Affiliations:** Department of Electrical & Computer Engineering, Rutgers University, Piscataway, NJ 08854, USA; yichao.yuan@rutgers.edu

**Keywords:** metamaterial (MTM), leaky wave antenna (LWA), radar sensor architecture, vital sign detection, location tracking

## Abstract

Microwave radar sensors have been developed for non-contact monitoring of the health condition and location of targets, which will cause minimal discomfort and eliminate sanitation issues, especially in a pandemic situation. To this end, several radar sensor architectures and algorithms have been proposed to detect multiple targets at different locations. Traditionally, beamforming techniques incorporating phase shifters or mechanical rotors are utilized, which is relatively complex and costly. On the other hand, metamaterial (MTM) leaky wave antennas (LWAs) have a unique property of launching waves of different spectral components in different directions. This feature can be utilized to detect multiple targets at different locations to obtain their healthcare and location information accurately, without complex structure and high cost. To this end, this paper reviews the recent development of MTM LWA-based radar sensor architectures for vital sign detection and location tracking. The experimental results demonstrate the effectiveness of MTM vital sign radar compared with different radar sensor architectures.

## 1. Introduction

**Figure 1:**
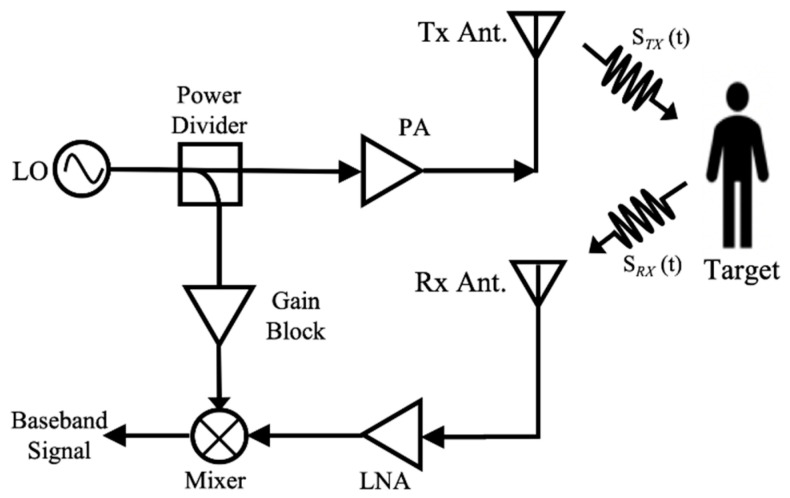
Block diagram of the homodyne radar sensor architecture.

Microwave radar sensors have been developed in physiological [1,2,3,4,5,6,7,8,9,10] or location tracking [11,12,13,14,15,16] of human targets over the past decades, with the development of semiconductors and algorithms. Owing to non-contact and penetrable characteristics of the radar sensor, this will cause minimal discomfort for the detected target and avoid the sanitation issues, which has gained much interest in the pandemic situation. Among them, the conventional homodyne radar sensor for vital sign detection illuminates the transmitted radiofrequency (RF) signal towards the target via the transmitting (Tx) antenna, while the reflected signal received by the receiving (Rx) antenna will be down-converted to the baseband signal through the mixer, as shown in Figure 1. Efforts have been made by researchers to improve the homodyne radar sensor architecture in recent years, in order to achieve accurate performance on vital sign detection. For example, quadrature Doppler radar receiver system is employed to solve the null point problem [17]. Furthermore, to mitigate the strong interference signal that is mainly caused by random body movement, a two-transceiver radar system with different polarizations and frequencies is placed at different body orientations to detect vital sign signals [18]. Another challenge for vital sign detection based on this architecture is 1/f noise, which can be overcome by low intermediate-frequency (IF) heterodyne radar sensor architectures [19].

Besides vital sign detection, microwave radar sensors can also be used for location tracking of the target. In [20], the distance to the target can be acquired by analyzing the direct current (dc) information associated with the target’s position from baseband signals with different beam-steering angles. On the other hand, a frequency-modulated continuous-wave (FMCW) modulation scheme is proposed to detect the distance to the target [21]. While the distance to the target can be obtained, directional information of targets is still a challenge for location tracking. To this end, mechanical rotors [22,23] and phased arrays [24] are proposed to detect different targets at different locations and obtain their directional information, respectively. Nonetheless, mechanical devices are bulky while phased arrays are complex and costly.

Recently, multiple-input multiple-output (MIMO) radar systems [25,26,27] have attracted considerable attention because of their capability for measuring angle-of-arrival (AOA) from the received signal, which is an indicator for the estimation of the target’s location with directional information. However, the implementation of multiple transceivers increases the systematic complexity and cost. As such, efforts have been made to reduce the systematic complexity. For instance, single-input multiple-output (SIMO) with different mechanisms, such as the digital beam forming (DBF) technique [28] or angle division multiplexing sensing (ADMS) [29], as well as the RF switch-based radar system [30] using a transceiver instead of a multiple radar system are employed to detect multiple targets. Although the number of transceivers can be effectively reduced, the radar systems still adopt multiple antennas at the transmitter or receiver end. Furthermore, the implementation of a control algorithm for the selection of corresponding antennas still increases the complexity of radar system.

**Figure 2:**
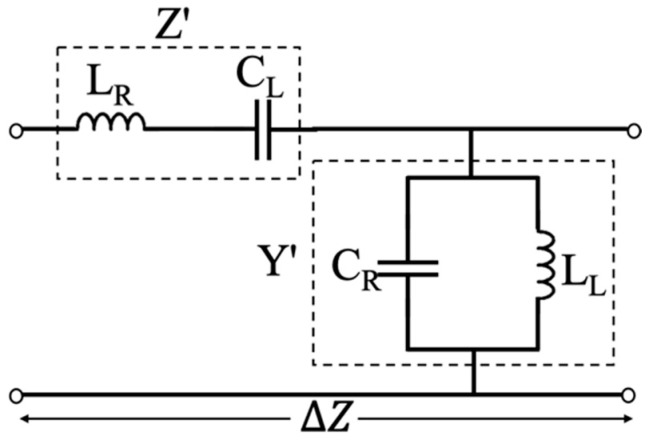
Equivalent Circuit of composite right/left-handed transmission line (CRLH TL) unit cell.

On the other hand, metamaterial leaky wave antennas (MTM LWAs) [31] exhibit a unique frequency to space mapping feature, which can be used to overcome the aforementioned shortcomings. In general, MTM is an artificial structure with unusual properties, such as negative index of refraction, which cannot be found in nature. It was first investigated by the Russian physicist Viktor Veselago in 1967, which he termed left-handed (LH) substance [32]. After more than 30 years, inspired by the work of J.B. Pendry et al., LH material was realized by Smith and colleagues at the University of California, San Diego (UCSD), consisting of copper split-ring resonators (SRRs) and thin copper wires, which exhibit negative permittivity (ε) and permeability (μ) [33]. In 2002, Caloz et al. [34] and Iyer et al. [35] proposed LH materials based on a transmission line (TL) structure. However, in practice, it is impossible to implement a purely LH TL structure because of unavoidable right-handed (RH) parasitic series inductance and shunt capacitance effects. Therefore, a composite right/left-handed (CRLH) structure is introduced in [31,36,37] to form MTM TLs. It is noted that the CRLH structure can also be realized based on the resonant-type approach [38], such as the use of split ring resonators (SRRs) or complementary split ring resonators (CSRRs). Based on the CRLH structure, MTM LWAs are shown to exhibit frequency-dependent beam-steering capability at the fundamental mode (*n* = 0) [39,40,41,42], which is different from other fundamentally slow-wave periodic structure-based LWAs operating at the higher spatial harmonics (|*n*| > 0) in order to generate fast waves, where *n* is an integer representing the order of spatial harmonic [43,44,45]. Figure 2 illustrates the equivalent circuit of a CRLH TL unit cell, which can exhibit backward-to-forward frequency-dependent beam scanning capability. Such characteristics have been widely used in antenna applications [46,47,48].

As shown in Figure 2, the CRLH TL unit cell consists of LH series capacitor (*C_L_*) and shunt inductor (*L_L_*) with parasitic RH series inductor (*L_R_*) and shunt capacitor (*C_R_*). Its general propagation constant can be obtained as:

(1) $$\gamma =j\beta =\frac{1}{\Delta z}\sqrt{Z^{\prime }Y^{\prime }},$$

where,

(2) $$Z^{\prime }=j\omega L_{R}+\frac{1}{j\omega C_{L}},\;\;Y^{\prime }=j\omega C_{R}+\frac{1}{j\omega L_{L}},$$

which leads to:

(3) $$\beta \left(\omega \right)=\frac{s\left(\omega \right)}{\Delta z}\sqrt{\omega^{2}L_{R}C_{R}+\frac{1}{\omega^{2}L_{L}C_{L}}-\left(\frac{L_{R}}{L_{L}}+\frac{C_{R}}{C_{L}}\right)},$$

where β(ω) is the phase constant of a TL, s(ω) and Δz are the sign function and the length of unit cell, respectively. For the balanced case, i.e., $L_{R}C_{L}=L_{L}C_{R}$, Equation (3) can be simplified as:

(4) $$\beta \left(\omega \right)=\beta_{RH}+\beta_{LH}=\frac{\omega }{\Delta z}\sqrt{L_{R}C_{R}}-\frac{1}{\omega \Delta z\sqrt{L_{L}C_{L}}},$$

**Figure 3:**
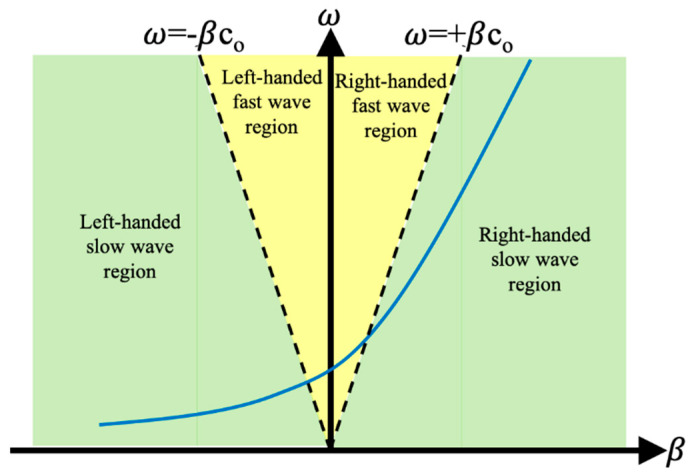
Dispersion diagram of CRLH TL (balanced case).

**Figure 4:**
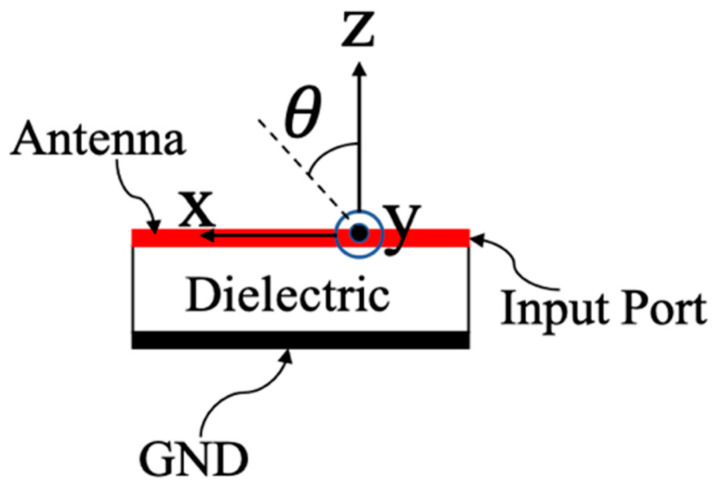
The radiation angle of CRLH TL.

Equation (4) shows the phase constant β(ω) can be changed from negative value to positive value as the frequency increases, which corresponds to the left-handed (LH) effects and right-handed (RH) effects. Its dispersion diagram is shown in Figure 3. In the fast wave region where the phase velocity is greater than the speed of light, radiation occurs with frequency-dependent radiation main beam angle θ for a CRLH TL given by Figure 4.

Specifically, the radiation angle of the main beam can be expressed as:

(5) $$\theta \left(\omega \right)={sin}^{-1}\left(\frac{\beta \left(\omega \right)}{k_{0}}\right)$$

where $k_{0}$ is the free space wave number. As the frequency varies, phase constant β(ω) of the CRLH TL is changed, which leads to the change of θ(ω). As a result, the CRLH MTM LWA is able to scan from the backfire to endfire direction, when β(ω) changes from negative to positive according to Equation (5). Owing to the frequency-dependent beam scanning capability of the MTM LWA, the target directional information can be easily found based on its radiation pattern and operational frequency.

The rest of this paper is organized as follows. Section 2 reviews homodyne architectures with one-dimensional (1D) MTM LWAs [49,50], which are employed for vital sign detection and location tracking based on the arctangent demodulation. Section 3 presents a self-injection-locked (SIL) radar architecture integrated with MTM LWAs to detect vital sign signals and track locations using frequency-shift keying (FSK) method [51]. Section 4 introduces a two-dimensional (2D) MTM LWA array for vital sign detection at 24 GHz band [52]. Finally, conclusions are made in Section 5.

## 2. Metamaterial Leaky Wave Antenna (MTM LWA)-Based Homodyne Architecture

**Figure 5:**
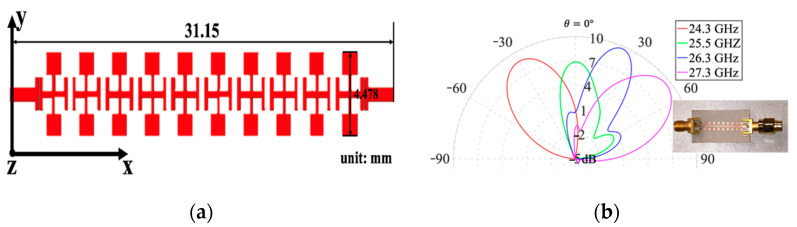
(**a**) Layout of 1D metamaterial leaky wave antenna (MTM LWA) at 24 GHz band; (**b**) its radiation pattern [49].

While conventional microwave radars have been widely studied for vital sign detection and location tracking of single target [17,53,54,55], the ability to perform multi-target detection has gained much interest recently [56,57]. Lu et al. applied a linearly polarized MTM LWA structure at 24 GHz band to detect the vital sign information of multiple targets and track their motions with homodyne architecture [49]. Figure 5 shows the layout and the radiation pattern of the 1D frequency-dependent beam scanning MTM LWA. In this design, a 1D frequency-dependent beam scanning MTM LWA consists of vialess unit cells with gap capacitors and open stub-loaded resonators, where *C_L_* is provided by a gap capacitor and *L_L_* is the inductance of an open stub, *L_R_* represents that parasitic inductance caused by current gradient flowing across gap capacitor, and *C_R_* is provided by the parallel plate effects between the signal layer and ground plane. According to its radiation pattern, the scanning angle of the MTM LWA can be changed from −30° to +50° as the operation frequency sweeps from 24.3 GHz to 27.3 GHz. It is noted that there is a 3 dB decrease in the radiation gain at the broadside, which may be avoided by forcing both series and shunt elements to contribute equally to the radiation [58,59].

**Figure 6:**
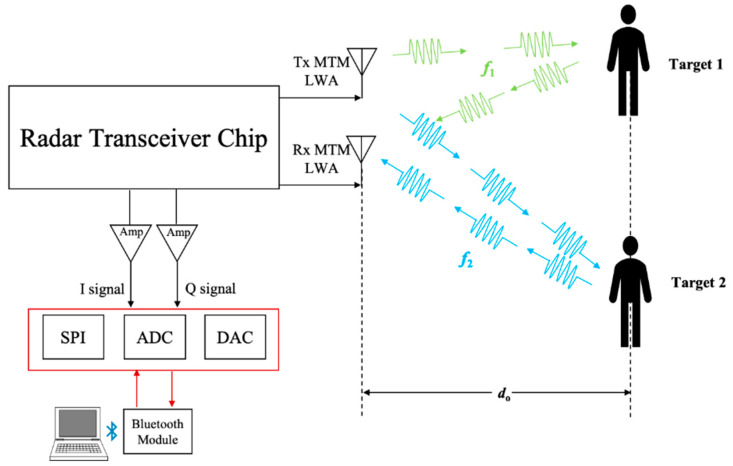
The block diagram of MTM LWA-based homodyne architecture at 24 GHz band.

The system block diagram of the proposed radar sensor module is shown in Figure 6. The radar transceiver chip is employed to control the operation frequency of MTM LWA. Consequently, based on its radiation pattern, the interrogating signal is transmitted via Tx MTM LWA towards the target along a specified direction, which can be expressed as:

(6) $$\theta \left(\omega \right)={sin}^{-1}\left(\frac{\beta \left(\omega \right)}{k_{0}}\right)$$

where *f* represents the operation frequency of MTM LWA and Φ(t) is the phase noise. The reflected signal, modulated by the target, is received by the Rx MTM LWA. Then it is down-converted to the I- and Q-baseband signals. A micro controller unit (MCU) digitalizes the signals, which are then transmitted through Bluetooth to the laptop for post processing. By normalizing the signal amplitude, the digitized I- and Q-baseband signals can be expressed as:

(7) $$B_{I}\left(n\right)=cos\left[\frac{4\pi d_{0}}{\lambda }+\frac{4\pi x_{v}\left(n\right)}{\lambda }+\theta_{0}+\Delta \Phi \right]+DC_{I}\left(n\right),$$

(8) $$B_{Q}\left(n\right)=sin\left[\frac{4\pi d_{0}}{\lambda }+\frac{4\pi x_{v}\left(n\right)}{\lambda }+\theta_{0}+\Delta \Phi \right]+DC_{Q}\left(n\right),$$

where $d_{0}$ is the nominal distance from the radar sensor to the target, $\theta_{0}$ accounts for the phase response due to the phase change at the target surface and phase delay between the antenna and mixer, ΔΦ represents the phase residue, and $DC_{I}\left(n\right)/DC_{Q}\left(n\right)$ are the dc offset of the in-phase and quadrature (I/Q) channels. Because $x_{v}\left(n\right)$ represents the displacement related to the vital sign signal of target, it can be denoted as:

(9) $$x_{v}\left(n\right)=A_{br}\cos\left(2\pi f_{br}t\right)+B_{h}\cos\left(2\pi f_{h}t\right),$$

where *A_br_*, *B_h_* are the displacements related to respiration and heartbeat activities and *f_br_*, *f_h_* are their corresponding frequencies. After calibrating the dc offset, the vital sign signals can be extracted in the frequency domain by performing fast Fourier transform (FFT) based on the arctangent demodulation method.

**Figure 7:**
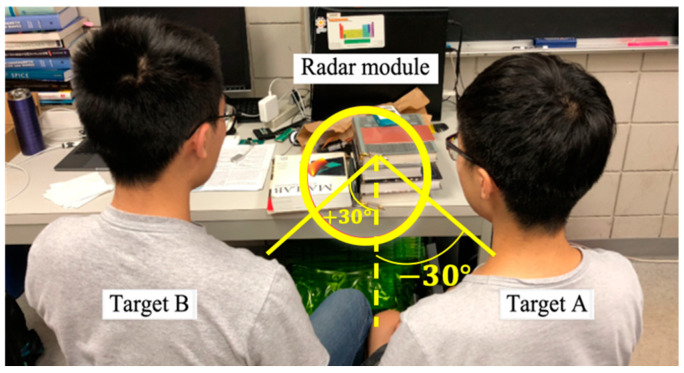
Experimental setup for detecting vital sign signals of two targets at different locations using MTM LWA-based homodyne radar sensor module at 24 GHz band [49].

In the experimental setup, shown in Figure 7, the vital sign signals of two targets sitting in front of the radar at different directions are chosen to be detected using the proposed radar sensor module. Target A sits at θ=−30° in the backward side and target B sits at θ=+30° in the forward side, in which the corresponding frequencies are 24.3 GHz and 26.3 GHz, according to the radiation patterns of MTM LWA. The distance to the targets is 0.8 m.

**Figure 8:**
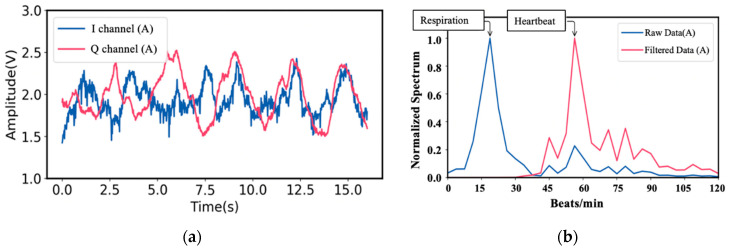
(**a**) Time domain vital sign signal of Target A; (**b**) the measured respiration and heartbeat rates of Target A using MTM LWA-based homodyne radar sensor module band [49].

**Figure 9:**
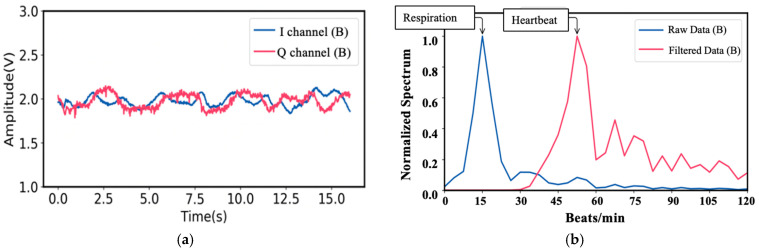
(**a**) Time domain vital sign signal of Target B; (**b**) the measured respiration and heartbeat rates of Target B using MTM LWA-based homodyne radar sensor module band [49].

For vital sign information of target A, the I- and Q-baseband signals in the time domain are shown in Figure 8a. As a result, corresponding respiration and heartbeat rate in the frequency domain are shown in Figure 8b. In the same way, the I- and Q-baseband signals for the vital sign information signal of Target B and his corresponding respiration and heartbeat rate are shown in Figure 9.

It is worth mentioning that a digital Butterworth high pass filter with the cutoff frequency of 0.5 Hz is employed to identify the heartbeat rate in the frequency domain in case the heartbeat information is overwhelmed by the harmonics coming from the stronger respiration signals. After performing FFT and filtering, the respiration and heartbeat rate of Target A are 18.6 beats/min and 56.4 beats/min, while the ground truth of the respiration and heartbeat rate of Target A are 17 beats/min and 58 beats/min, respectively. On the other hand, for Target B, the respiration and heartbeat rate are 15 beats/min and 56 beats/min, while the ground truth are 15 beats/min and 59 beats/min, respectively.

In addition to the vital sign signal detection, the target motion can also be tracked by using MTM LWA-based homodyne radar sensor module [50]. According to Equations (7) and (8), the I- and Q-baseband signals incorporate distance information between radar sensor and the target. Therefore, by calibrating the dc offset, the I- and Q-baseband signals can be expressed as:

(10) $$B_{I}\left(n\right)=cos\left[\frac{4\pi d_{0}}{\lambda }+\frac{4\pi x_{v}\left(n\right)}{\lambda }+\theta_{0}+\Delta \Phi \right],$$

(11) $$B_{Q}\left(n\right)=sin\left[\frac{4\pi d_{0}}{\lambda }+\frac{4\pi x_{v}\left(n\right)}{\lambda }+\theta_{0}+\Delta \Phi \right],$$

By applying the arctangent demodulation method [17], the phase information of baseband signals can be extracted as:

(12) $$\Phi \left(n\right)=tan^{-1}\left(\frac{B_{Q}\left(n\right)}{B_{I}\left(n\right)}\right)=\frac{4\pi d_{0}}{\lambda }+\frac{4\pi x_{v}\left(n\right)}{\lambda }+\theta_{0}+\Delta \Phi ,$$

where the small movement $x_{v}\left(n\right)$ can be neglected compared with the distance $d_{0}$, and Δϕ can be ignored due to the range correlation theory with a small detection displacement [60]. Assuming $\theta_{0}$ is a constant for the target, the relative distance $\Delta d=d_{0}\left(n_{1}\right)-d_{0}\left(n_{2}\right)$ can be obtained based on Equation (13):

(13) $$\Delta d\approx \frac{\lambda \cdot \Delta \Phi \left(n\right)}{4\pi },$$

**Figure 10:**
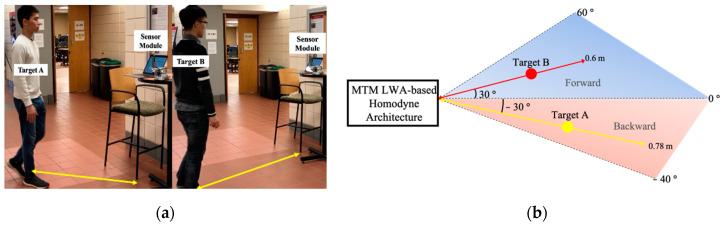
(**a**) Experimental setup for multi-target motion detection using MTM LWA-based homodyne radar sensor module; (**b**) its top view sketch.

The experimental setup of the multi-target motion detection is shown in Figure 10a. The main beam of the radar sensor illuminates two human targets with different operation frequencies. In this case, Target A moves back and forth with directional angle at θ=−30° in the backward side of the MTM LWA and Target B moves back and forth with directional angle at θ=+30° in the forward side of the MTM LWA. The top view sketch is shown in Figure 10b, where the corresponding operation frequencies of MTM LWA are 24.3 GHz and 26.3 GHz, respectively.

**Figure 11:**
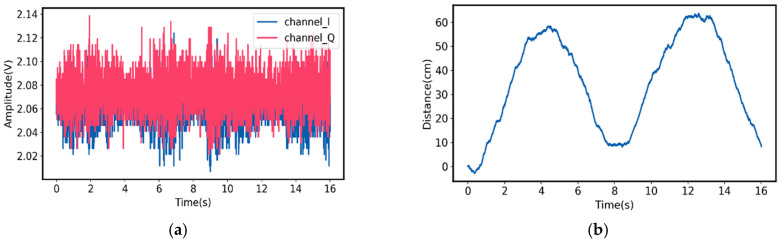
(**a**) Measured I- and Q-baseband signals for Target A motion at 24.3 GHz; (**b**) measured relative moving distance of Target A [50].

**Figure 12:**
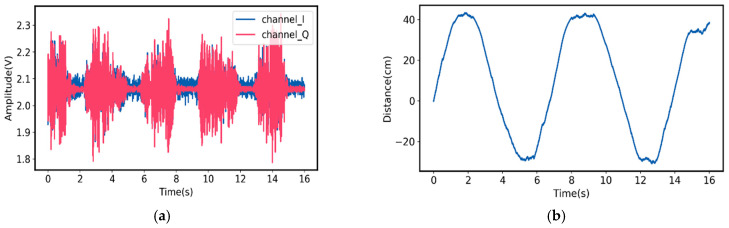
(**a**) Measured I- and Q-baseband signals for Target B motion at 26.3 GHz; (**b**) measured relative moving distance of Target B [50].

The I- and Q-baseband signals for Target A motion and the measured relative moving distance, based on Equation (13), are shown in Figure 11. For Target B, the I- and Q-baseband signals and its measured relative moving distance are plotted in Figure 12. As can be seen, for Target A, the measured relative moving distance is from 0 to 63 cm, while the ground truth is from 0 to 60 cm, leading to 5% deviation. On the other hand, for Target B, the measured relative moving distance is from 0 to 75 cm and the ground truth is from 0 to 78 cm, which only leads to 3.8% deviation.

## 3. MTM LWA Based on Self-Injection Locked (SIL) Radar Architecture

**Figure 13:**
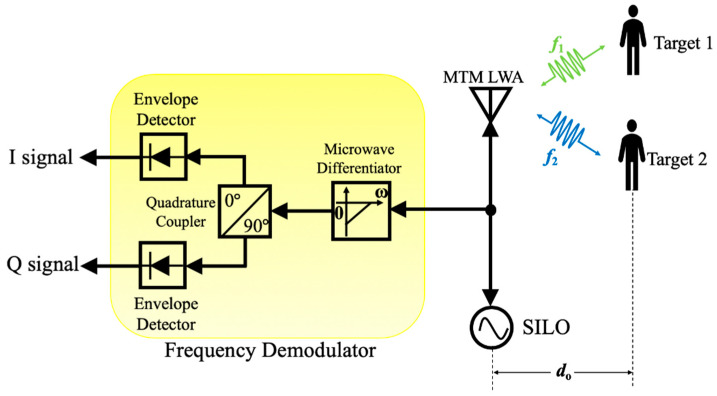
The block diagram of proposed MTM LWA based on self-injection-locked (SIL) radar sensor architecture, illuminating two distinct operation frequencies to two targets.

In addition to conventional radar sensor architectures, vital sign radar detection based on self-injection locked (SIL) architecture has been proposed recently [61], which has gained much attention because of its low system complexity and high sensitivity [61,62]. Yuan et al. [51] proposed a new architecture of SIL radar sensor integrated with an MTM LWA, where its block diagram is shown in Figure 13. In this scenario, the interrogating RF signal with free-running oscillation frequency, $\omega_{osc}$, is illuminated via an MTM LWA coming from a self-injection locked oscillator (SILO). The reflected signal modulated by the target locks the oscillator into SIL state. Therefore, the output frequency $\omega_{out}\left(t\right)$ of the SILO will be injection-locked to a different value. Based on Adler’s equation [62], it can be expressed as:

(14) $$\omega_{out}\left(t\right)=\omega_{osc}-\omega_{LR}cos\alpha \left(t\right),$$

where $\omega_{LR}$ denotes as the locking range and α(t) is phase delay of SIL path, which can be given by:

(15) $$\omega_{LR}=\frac{\omega_{osc}}{2Q_{tank}}\frac{E_{inj}}{E_{osc}},$$

(16) $$\alpha \left(t\right)=\frac{2\omega_{osc}}{c}\left(d_{o}+x_{v}\left(t\right)\right),$$

where $Q_{tank}$ is the quality factor of SILO’s tank circuit, $E_{inj}$ is the amplitude of injection signal and $E_{osc}$ is the amplitude of the free-running signal of the SILO. Therefore, the frequency-modulated output signal of MTM LWA is:

(17) $$S_{out}\left(t\right)=A_{out}cos\left(\omega_{out}t\right),$$

where $A_{out}$ is the amplitude of output signal. The signal will then be sent into a first-order microwave differentiator to convert the frequency modulation (FM) of signal to an amplitude modulation (AM) signal, which is given by:

(18) $$\frac{dS_{out}\left(t\right)}{dt}=-\omega_{out}A_{out}sin\left(\omega_{out}t\right),$$

A quadrature coupler followed by two envelope detectors is then utilized to perform AM demodulation for the I/Q channel. The vital sign signals with distance information of multiple targets at different locations can be obtained by performing FFT of the demodulated I/Q signals.

**Figure 14:**
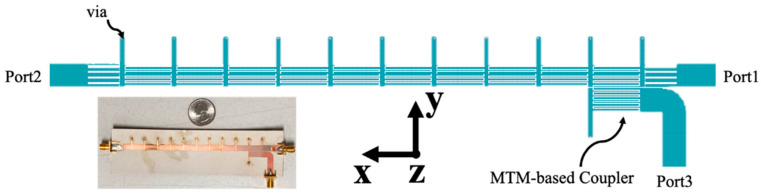
Layout and prototype of 1-D MTM LWA at 2.4 GHz.

**Figure 15:**
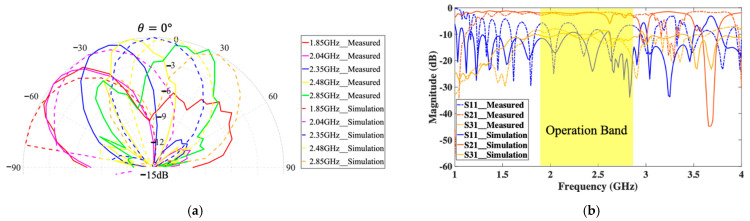
(**a**) Radiation pattern of 1-D MTM LWA at 2.4 GHz; (**b**) its S-parameters [51].

To detect multiple targets at different locations, a 1D linearly polarized frequency-dependent beam scanning MTM LWA with MTM-based coupler operating at the 2.4 GHz band is designed. Its layout and prototype are shown in Figure 14, which consists of 10 CRLH unit cells. Based on its radiation pattern shown in Figure 15a, the scanning angle of MTM LWA can be changed from −50° to +30° when the operation frequency varies from 1.85 GHz to 2.85 GHz. The reflection coefficient (*S*_11_) is close to −10 dB within the same operation frequency range, as shown in Figure 15b.

**Figure 16:**
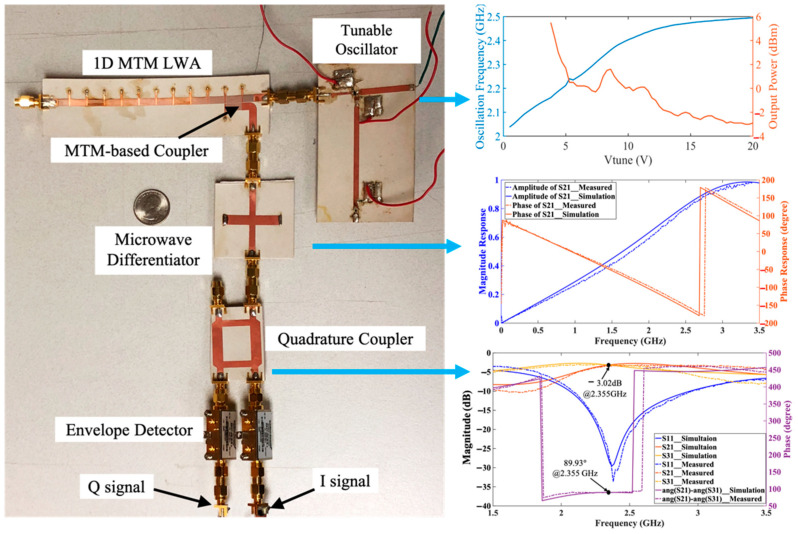
Prototype of the MTM LWA based on SIL radar sensor architecture at 2.4 GHz band.

Figure 16 presents the prototype of the radar sensor module. The operation frequency of MTM LWA can be tuned from 2.04 GHz to 2.48 GHz through a tunable oscillator, which is designed based on the negative resistance method [63] with common source configuration. The oscillator output is connected to Port 1 of the MTM LWA integrated with the MTM-based coupler to transmit and receive RF signals. When the modulated signal within the frequency band of 2.04 GHz to 2.48 GHz is received by MTM LWA, it will be sent through the MTM-based coupler to the first-order microwave differentiator for frequency demodulation [64], whose magnitude response is linear with respect to the frequency from dc to 3.1 GHz [65]. Consequently, the modulated signal can be converted to the RF signal with the corresponding frequency-dependent amplitudes. Then a quadrature coupler followed by the two envelope detectors is included to demodulate the frequency dependent amplitude signal into I/Q signals for further post processing.

**Figure 17:**
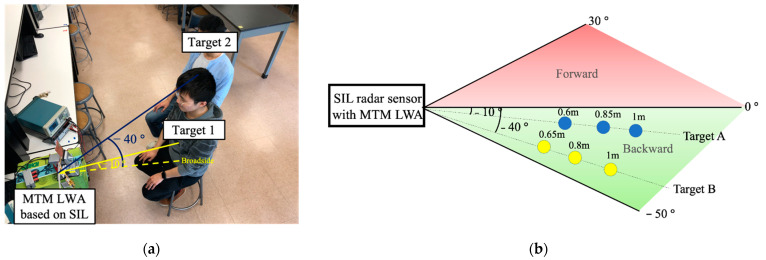
(**a**) Experimental setup for detecting vital sign signals of two targets at different locations using MTM LWA based on SIL radar sensor module; (**b**) its top view sketch [51].

**Figure 18:**
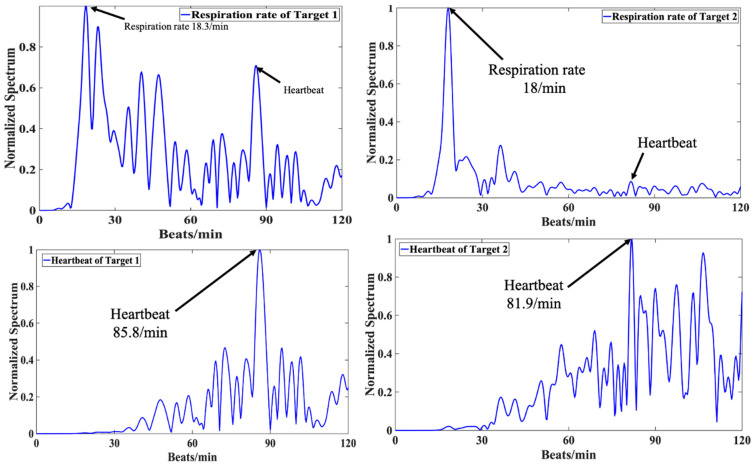
Measured respiration and heartbeat rates of Target 1 and 2 using MTM LWA based on SIL radar sensor module [51].

To validate the proposed MTM SIL radar, the experimental setup and its top view sketch are shown in Figure 17. In Figure 17a, Target 1 is located at a distance of 0.6 m with θ=−10°, whereas Target 2 is located at a distance of 0.65 m with θ=−40° in the backward side. The breath rate and heartbeat incorporated with a high pass filter at the cutoff frequency of 0.5 Hz for both targets are presented in Figure 18. The measured respiration rates for target 1 and target 2 are 18.3 beats/min and 18 beat/min, while the ground truth for both targets is 18 beats/min. The heartbeat rates for target 1 and 2 are 85.8 beats/min and 81.9 beats/min, while the ground truth are 87 beats/min and 81 beats/min for both targets, respectively.

**Figure 19:**
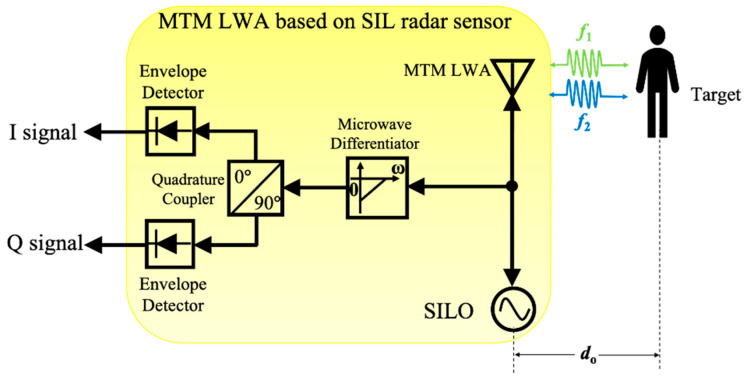
Frequency-shift keying (FSK) modulated SIL radar sensor for concurrent vital sign detection and location tracking of multiple targets.

The proposed MTM SIL radar sensor can track target location as well as detecting vital sign signals concurrently. For location tracking, the angular information of targets can be obtained from the dispersion relation based on the radiation pattern of MTM LWAs [47,66]. To further obtain the distance to the target, based on the frequency-shift keying (FSK) method [67,68], two closely adjacent carrier frequencies *f*_1_ and *f*_2_ with a small frequency difference $\Delta f=f_{2}-f_{1}$ are chosen to illuminate the target, presented in Figure 19. In this case, the radiation angle θ for *f*_1_ and *f*_2_ are assumed to be the same, due to the small frequency difference.

To illustrate the method, the transmitted signal is represented as:

(19) $$T_{k}\left(t\right)=\cos\left[2\pi f_{k}t+\Phi_{k}\left(t\right)\right],$$

where *k* = 1, 2 and $\phi_{k}\left(t\right)$ are the phase noise from the voltage-controlled oscillator (VCO). When a human target is located at the distance of $d_{0}$, the reflected signal with phase including the distance information, is injected into the oscillator. Neglecting the amplitude response for simplicity, it can be expressed as

(20) $$R_{k}\left(t\right)=\cos\left[2\pi f_{k}t-\frac{4\pi (d_{0}+x_{v}\left(t\right))}{\lambda_{k}}+\Phi_{k}\left(t-\frac{2d_{0}}{c}\right)\right],$$

where $x_{v}\left(t\right)$ represents the cardiac activity. According to Equation (19), because $x_{v}\left(t\right)$ is much smaller than the distance $d_{0}$, the phase term is approximately:

(21) $$\theta_{k}\left(t\right)=\frac{4\pi (d_{0}+x_{v}\left(t\right))}{\lambda_{k}}\approx \frac{4\pi d_{0}}{\lambda_{k}},$$

**Figure 20:**
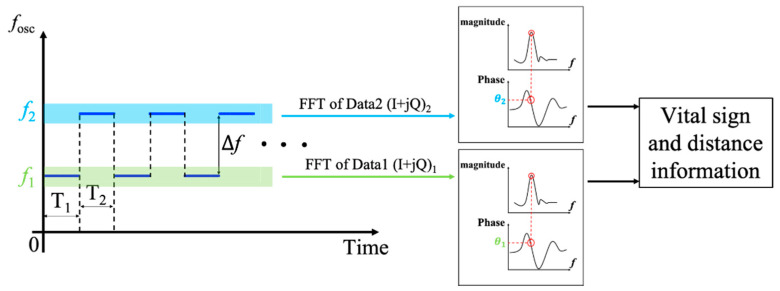
The block diagram of FSK signal processing under pulse-width modulation (PWM) control.

Therefore, by imposing the FSK method under pulse-width modulation (PWM) control, where the block diagram is shown in Figure 20, the phase difference of the two adjacent tones is:

(22) $$\Delta \theta =\theta_{2}-\theta_{1}=\frac{4\pi d_{0}}{\lambda_{2}}-\frac{4\pi d_{0}}{\lambda_{1}}=\frac{4\pi \Delta fd_{0}}{c},$$

As such, the distance between the target and radar sensor can be calculated using:

(23) $$d_{0}=\frac{c\Delta \theta }{4\pi \Delta f},$$

**Figure 21:**
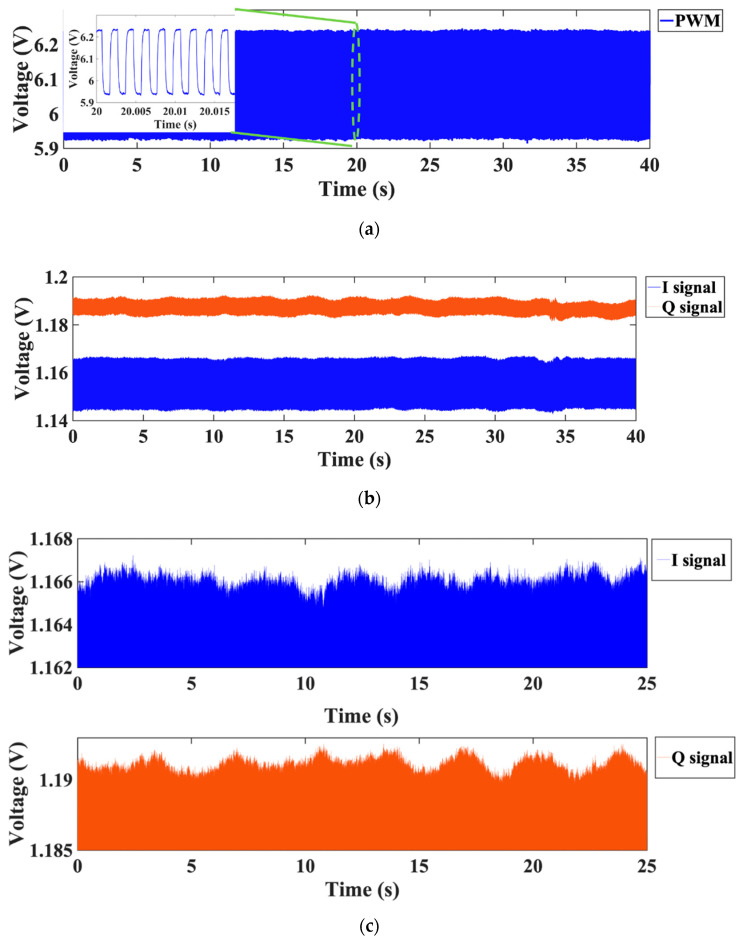
(**a**) Measured PWM control signal; (**b**) measured time domain I/Q signals and (**c**) their zoomed-in view of the target located at the distance of 0.65 m [51].

**Figure 22:**
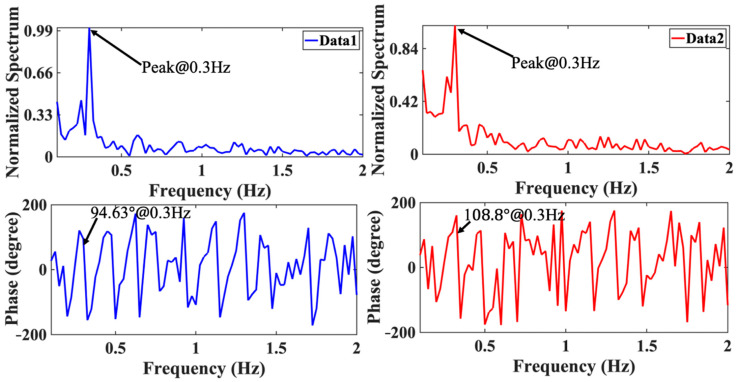
Normalized spectrum with phase information of the target located at the distance of 0.65 m.

If *f*_1_ and *f*_2_ are switched repeatedly at a small time interval of *T*_1_ and *T*_2_, respectively, *f*_1_ and *f*_2_ can be considered to work simultaneously. Meanwhile, because of the small frequency difference Δf, their frequency spectrums under the same Doppler frequency have almost the same amplitude response, but different phase responses. Consequently, once the phase difference Δθ is obtained through executing the FFT, the distance can be calculated from (23). Figure 21 depicts the I- and Q-baseband signals and their zoomed-in view in the time domain with the PWM control signal, when the target is at the distance of 0.65 m. It is noted that the periodic amplitude change can be observed clearly, which is related to the chest motion due to the respiratory activity of the human target. The normalized spectrum and phase information of the target are presented in Figure 22.

The measured breath rate is 18 beats/min, which agrees well with the ground truth. Meanwhile, the phase response at the Doppler frequency, *f* = 0.3 Hz, is utilized to calculate the distance through Equation (23), which gives 0.7 m with the actual distance of 0.65 m. Along with the angular information obtained from the radiation pattern of the MTM LWA, the target location in 2D space can be obtained. In Figure 17b, when both targets sit at different locations in front of the radar sensor, the mean values of calculated distance with corresponding respiration and heartbeat rate are summarized in Table 1 and Table 2, respectively.

## 4. Two-Dimensional (2D) Beam Scanning Metamaterial LWA Design

**Figure 23:**
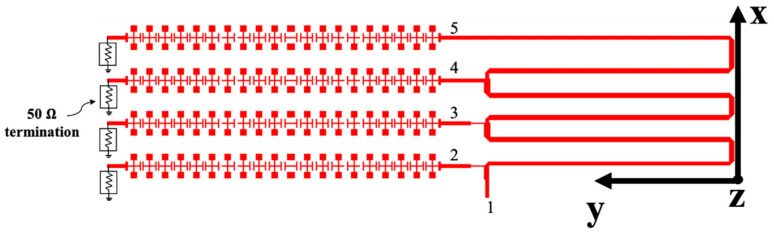
Layout of 2D MTM LWA array at 24 GHz band [52].

**Figure 24:**
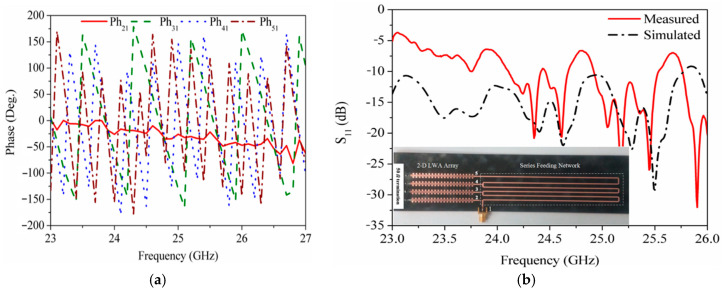
(**a**) Phase responses of the series feeding network; (**b**) S11 of 2D frequency-dependent beam scanning MTM LWA array [52].

**Figure 25:**
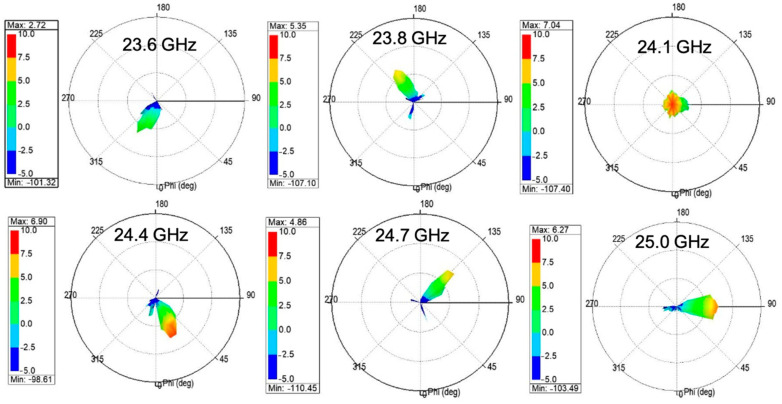
Radiation pattern of 2D MTM LWA array at 24 GHz band [52].

As indicated in (5), the CRLH TL can be applied to fabricate the MTM LWA to achieve the frequency-dependent beam scanning capability. To realize the 2D beam scanning for vital sign detection, a 2D MTM LWA array operating at 24 GHz band is proposed [52], which incorporates four 1D frequency-dependent beam scanning MTM LWAs with a series feeding network, shown in Figure 23. The series feeding network contributes to the different phase responses for four 1D frequency-dependent beam scanning MTM LWAs at one operation frequency, illustrated in Figure 24a. Therefore, 2D beam scanning can be realized by controlling the phase difference between each 1D antenna element. Figure 24b shows the reflection coefficient (*S*_11_) is less than −6 dB from 23.4 to 25.4 GHz. Within the same operation frequency range, its radiation pattern is shown in Figure 25. It can be seen that the main beam scans in a 2D fashion with respect to the frequency change.

**Figure 26:**
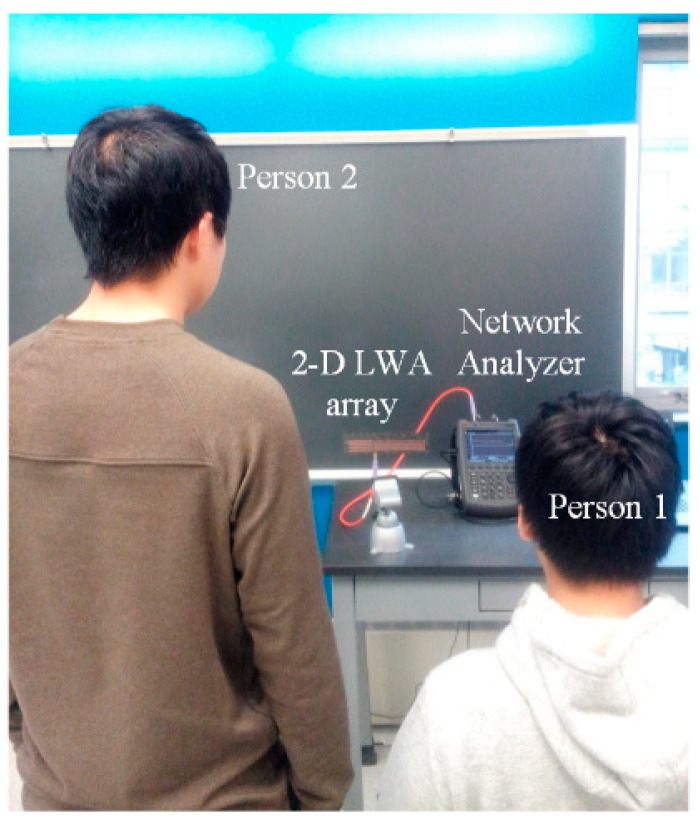
Experimental setup for multiple targets with different heights using 2D frequency-dependent beam scanning MTM LWA array [52].

**Figure 27:**
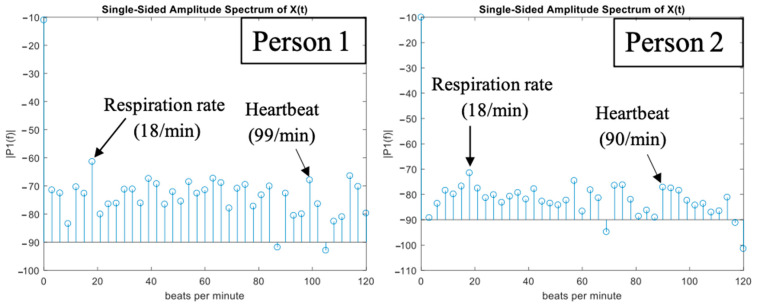
The measured respiration and heartbeat rates of two targets [52].

As such, the designed 2D frequency-dependent beam scanning MTM LWA array can be used to detect the vital sign signals of multiple targets. To verify the 2D beam scanning capability, the designed MTM LWA array connected with a vector network analyzer (VNA) is utilized to detect vital sign signals of two persons with different heights, as shown in Figure 26. In this scenario, Person 1 sits with his chest facing the radiation angle of the main beam at 24.1 GHz, while Person 2 stands with his chest facing the radiation angle of the main beam at 24.4 GHz. By performing the FFT on the scattering parameters obtained from the VNA, vital sign signals, including respiration and heartbeat rates, can be obtained as shown in Figure 27. The measured respiration and heartbeat rates of Person 1 are 18 beats/min and 99 beats/min, respectively. Meanwhile, the measured respiration and heartbeat rates of Person 2 are 18 beats/min and 90 beats/min, respectively.

## 5. Conclusions

Over the past few decades, vital sign detection and location tracking using radar sensor modules have become a hot topic because of their non-contact and penetrable characteristics. With the development of semiconductors and algorithms, it has been demonstrated that different radar sensor architectures can obtain accurate vital sign information and target locations. On the other hand, multi-target detection based on radar sensor module is likely to be a trend for detecting vital sign information and location tracking. The main properties of different methods for multi-target detection using radar sensors are summarized in Table 3. As can be seen, MTM LWA-based vital radar sensors have several advantages such as low complexity and compact size. To this end, this paper reviews the recent development of radar sensors based on frequency-dependent beam scanning using MTM LWA, including MTM LWA-based homodyne architecture, MTM LWA using the self-injection locked (SIL) radar architecture, and 2D beam scanning MTM LWA design. Experimental detection results reveal the effectiveness of vital sign signals and location tracking for multiple targets using MTM LWAs based on these architectures.

## Figures and Tables

**Table 1:** Calculated distance results for both targets.

	Mean of the Calculation Results (m)	Ground Truth (m) ^1^	Error (m)	Standard Deviation (m)
**Target 1**	0.55	0.6	0.05	0.03
	0.81	0.85	0.04	0.04
	1.01	1	−0.01	0.02
**Target 2**	0.7	0.65	0.05	0.08
	0.77	0.8	0.03	0.06
	0.98	1	0.02	0.04

^1^ The uncertainty of the ground truth for distance is 0.08 m.

**Table 2:** Vital sign results for both targets.

	Distance (m)	Respiration (beats/min)	Ground Truth	Heartbeat (beats/min)	Ground Truth
**Target 1**	0.6	18.3	18	85.8	87
	0.85	18	17	86.7	86
	1	16.5	17	75	77
**Target 2**	0.65	18	18	81.9	81
	0.8	15	15	83.4	83
	1	15	16	79	80

**Table 3:** Main properties of methods for multi-target detection using radar sensors.

Radar Configuration	Signal Processing Method	Number of Antennas Used	System Size
Mechanical Rotors [23]	FFT	Low	Large
SIMO [29]	ADMS etc.	High	Medium
MIMO [26]	Frequency analysis etc.	High	Medium
RF switch-based radar system [30]	Heuristic digital signal processing	High	Medium
MTM LWA [51]	FFT	Low	Small

## Data Availability

The data presented in this study are available on request from the corresponding author.
